# Towards Genetically Informed Conservation of the Bardoka and Karakachan Sheep Breeds Autochthonous to Serbia

**DOI:** 10.3390/ani15091193

**Published:** 2025-04-23

**Authors:** Ivan Ćosić, Krstina Zeljić Stojiljković, Ivan Pihler, Bogdan Cekić, Dragana Ružić-Muslić, Nikola Delić, Jelena M. Aleksić

**Affiliations:** 1 Institute for Animal Husbandry, Belgrade-Zemun, Autoput 16, P.O. Box 23, 11080 Belgrade, Serbia; ivancosic58@yahoo.com (I.Ć.); krstina.zeljic@yahoo.com (K.Z.S.); bcekic@istocar.bg.ac.rs (B.C.); muslic.ruzic@gmail.com (D.R.-M.); delicnikola68@yahoo.com (N.D.); 2 Department of Animal Science, Faculty of Agriculture, University of Novi Sad, Trg Dositeja Obradovića 8, 21000 Novi Sad, Serbia; ipihler@gmail.com; 3 Institute for Medical Research, National Institute of Republic of Serbia, University of Belgrade, Bulevar oslobođenja 18, 11000 Belgrade, Serbia

**Keywords:** autochthonous sheep breeds, genetic diversity, genetic differentiation, inbreeding, nuclear microsatellites, relatedness, the Balkans, Serbia, Yugoslav Zackel

## Abstract

Bardoka and Karakachan sheep are primitive regional transboundary Pramenka-type sheep autochthonous to the Balkan Peninsula, whose populations have been reduced to a critically small size in Serbia. We genotyped 105 Bardoka animals (97 ewes and 8 rams from three flocks) and 97 Karakachan sheep (86 ewes and 11 rams from four flocks), along with 28 Ile-de-France (IDF) animals used for a comparison (25 ewes and 3 rams), using 14 nuclear microsatellites to assess their genetic status and establish a foundation for their genetically informed conservation. We utilized genetic data to assess inbreeding values of individuals (I) and pairwise relatedness (r) traditionally inferred from the pedigree data, which are incomplete in the studied autochthonous breeds. We used these data to assemble a data set of unrelated individuals for subsequent genetic analyses. Low but statistically significant genetic differentiation of Bardoka and Karakachan sheep (F_ST_ = 0.031, *p* < 0.01) demonstrates that these phenotypically distinct breeds differ at the genetic level as well. The I and r in ewes were higher in the Karakachan sheep (I = 0.09, r = 0.07) than in Bardoka (I = 0.06, r = 0.06). Contrary to the IDF rams, Bardoka and Karakachan sheep rams were genetically heterogeneous and those of the latter breed displayed higher average r values (0.01 vs. 0.08, respectively). Rams of both local breeds had identical I values of 0.02. Although Bardoka and Karakachan sheep still harbor rather high levels of genetic diversity (H_E_ = 0.761 ± 0.028 and 0.761 ± 0.021, respectively), the overall genetic data demonstrate that the genetic consequences of the population decline were more severe in the latter breed. A genetic structure presenting a general trend of differentiation of flocks with low genetic exchange into separate genetic entities was observed, indicating the effects of genetic drift. The implementation of the genetically informed conservation, together with the ongoing efforts of the state to enlarge the Bardoka and Karakachan sheep populations, would increase the prospects for the long-term survival of both breeds in Serbia.

## Simple Summary

Locally adapted sheep populations that are typically characterized by low productivity are currently valued not only for their contribution to food security in rural areas, but also as living reservoirs of valuable traits that can potentially be used to improve mainstream breeds. When these sheep populations decline, their survival is mainly threatened due to inbreeding, which has negative effects on sheep reproduction rates and productivity. We use molecular markers to characterize a large proportion (>50%) of living animals of critically endangered Bardoka and Karakachan sheep from Serbia, in order to gain knowledge of their current genetic status and to provide a basis for their genetically informed conservation. Our data reveal that these phenotypically distinct breeds differ at the genetic level as well, and demonstrate rather high levels of genetic diversity in both breeds and their genetic structure. However, population size decline has had a more profound negative effect on the ewes and rams of the Karakachan sheep than on those of the Bardoka. Our genetic data can be used for the improvement of management practices and the implementation of genetically informed conservation in the studied breeds which, together with the current efforts of the state to enlarge Bardoka and Karakachan sheep populations, increase the prospects for their long-term survival.

## 1. Introduction

The domestic sheep *Ovis aries* L. and the domestic goat *Capra hircus* L. are among the first domesticated livestock species (ca. 10,000 years ago in the Fertile Crescent) [1,2]. The Balkan Peninsula served as the major entry point of domesticated sheep into Europe during the Neolithic and as the post-domestication migration hub for their subsequent expansion throughout the continent [1,2,3]. This region is characterized by abundant mountainous and semi-mountainous regions with diverse geomorphological and climatic conditions that are particularly suitable for pastoral farming and sheep grazing [4] which, together with a variety of diverse sociocultural conditions, have facilitated the development of numerous local sheep populations in the Balkans over time [5]. At present, autochthonous Balkan sheep breeds exhibit considerable genetic overlap but are clearly distinct from the local breeds in surrounding regions [3], and are referred to as a “genetic hot spot” due to their high levels of genetic diversity [3,6,7]. The conservation of such breeds is rather important as locally adapted sheep populations contribute not only to food security in rural areas, but also serve as living reservoirs of biodiversity with genetic adaptive fitness traits that can be used to improve mainstream agriculture [8].

Pramenka is a primary type of domestic sheep from which, under the influence of various biogeographical and sociocultural conditions, historically numerous local breeds of sheep have emerged [5,9,10,11,12]. Extant Pramenka-type breeds from the Balkans are well adapted to extensive management in extreme climate conditions and rearing on pastures that are less favorable for commercial breeds [5,11,12]. They are typically characterized by low productivity but are currently valued for features such as resistance to diseases, longevity, pronounced maternal instinct, ability to walk over long distances, and grazing on poorly accessible terrain [5,11,12]. These, together with their historic, socioeconomic, and cultural importance, further support the requirement for their well-defined conservation and sustainable use [13,14,15,16]. Recent genetic studies of various Pramenka-type sheep breeds from the Balkans [17,18,19] highlight certain shortcomings of the past breeding and management practices relevant for their conservation. The most prominent shortcomings are intentional or unintentional uncontrolled crossbreeding as well as periodic declines in population size. While the former generally affects genetic integrity of the breeds, the latter contributes towards genetic drift and increased inbreeding [20,21,22]. Inbreeding is known for its negative effects on the reproduction rate, productivity, and survival [23,24,25]. Increased inbreeding results in the loss of genetic diversity, which hampers the ability of populations to cope with environmental changes [26,27].

Ten Pramenka-type breeds, namely Balusha, Bardoka, Karakachan sheep, Krivovirska, Lipska, Pirotska, Sharmountain, Sjenica, Svrljig and Vlach-vitohorn (syn. Racka sheep and Valachian sheep), are currently present in Serbia. Based on their extant total population size, they are classified into appropriate categories of risk status based on the FAO criteria [28]. In Serbia, these Pramenka-type breeds have been studied to date almost exclusively with respect to their phenotype [29,30,31] and productivity [32,33,34], and rarely at the molecular level [13,35,36]. However, genetic/genomic data are needed, because they may serve, among other aspects, as a powerful tool for assessing relatedness and inbreeding values of individuals in cases when the pedigree data are incomplete and/or missing for various reasons [37,38,39]. In addition, they can be used for setting rules for management practice, similar to what, for example, Ramljak et al. (2018) [40] accomplished in the case of the regional busha metapopulation. The implementation of such rules in conservation programs would lead to genetically informed conservation of autochthonous breeds, which would greatly increase the prospects for their long-term survival [40].

The aim of our study was to set the grounds for the genetically informed conservation of two Pramenka-type breeds present in Serbia, namely Bardoka and Karakachan sheep, which are critically endangered due to the low number of living animals [15,41,42]. To this end, we use 14 nuclear microsatellites recommended by the International Society for Animal Genetics (ISAG) and the Food and Agriculture Organization of the United Nations (FAO UN) for sheep genotyping to analyze 230 animals belonging to Bardoka (97 ewes and 8 rams from three flocks), Karakachan (86 ewes and 11 rams from four flocks) and 28 Ile-de-France (IDF) animals used for the comparison (25 ewes and 3 rams). We utilized molecular data to assess the relatedness of all animals at the population level as well as inbreeding values of individuals that are traditionally inferred from the pedigree data (which are incomplete for Bardoka and Karakachan sheep), in order to assemble a data set of unrelated individuals used for all genetic analyses. We then undertook the following: (i) assessing the levels of genetic diversity in each flock and each breed; (ii) assessing the levels of genetic differentiation of flocks within breeds and between breeds; and (iii) assessing the genetic layout of ram populations in both autochthonous breeds and estimating their suitability for breeding, with the aim of maintaining levels of genetic diversity and avoiding inbreeding.

## 2. Materials and Methods

### 2.1. Ethical Statement and Animal Welfare

All experimental procedures conducted in this study were reviewed and approved by the Ethics Committee of Veterinary Administration of the Ministry of Agriculture, Forestry and Water Management of the Republic of Serbia (protocol code 323-07-06185/2021/05, 17 September 2021). During the sampling, we took care of animal welfare.

### 2.2. Characteristics of the Studied Pramenka Sheep

**Figure 1:**
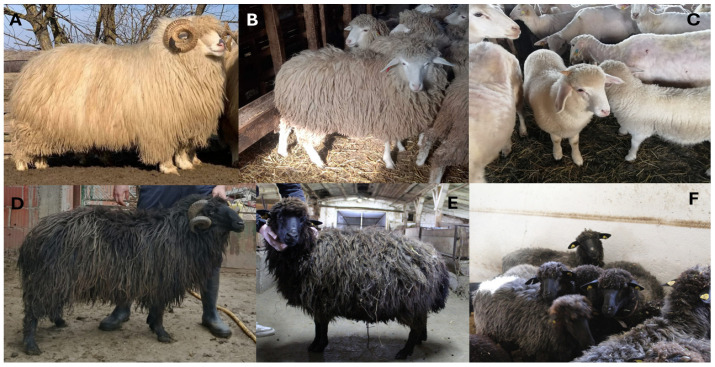
Phenotypes of Bardoka and Karakachan sheep. (**A**) Bardoka ram, (**B**) Bardoka ewe, (**C**) Bardoka flock, (**D**) Karakachan sheep ram, (**E**) Karakachan sheep ewe, and (**F**) Karakachan sheep flock. Photographs by I. Ćosić, B. Cekić, A. Vasov, and V. Nikolic.

Bardoka and Karakachan sheep (Figure 1) are small, hardy, primitive, thin-tailed transboundary Pramenka-type sheep breeds [5,11,12]. Their typical morphological feature is coarse wool with open fleece [5,11,12]. Based on their extant low population size in Serbia, they are characterized as critically endangered following the FAO risk status criteria [28].

#### 2.2.1. Bardoka

Bardoka is currently present in Albania, Kosovo and Metohija, Serbia, and Montenegro [10], where the breed is used for combined production traits (milk, meat, and wool). Bardoka sheep are slightly larger than Karakachan sheep, and their typical phenotype is shown in Figure 1A–C. In Bardoka rams, the height at withers is 70–75 cm, and live weight is 75–85 kg, while in ewes, the height at withers is 60–65 cm, and live weight is 50–55 kg [11,12,43]. Bardoka wool is coarse, up to 30 cm long, and typically white. The fleece is open and composed of spiky locks made of very coarse fibers that do not curve [11,12]. The wool is of low density, covers all body parts including the head, and forms a tuft between the ears, while the ears and legs are covered with white hair. The head is short, broad, and flat in profile. Rams have strong, spaced horns with a triangular cross-section while ewes are always hornless. Bardoka is one of the highest milk-yielding Pramenka-type breeds with an average milk yield of ~110 kg (occasionally up to 200 kg) per lactation, partly due to prolonged lactation, which can last over 7 months [11,12].

**Figure 2:**
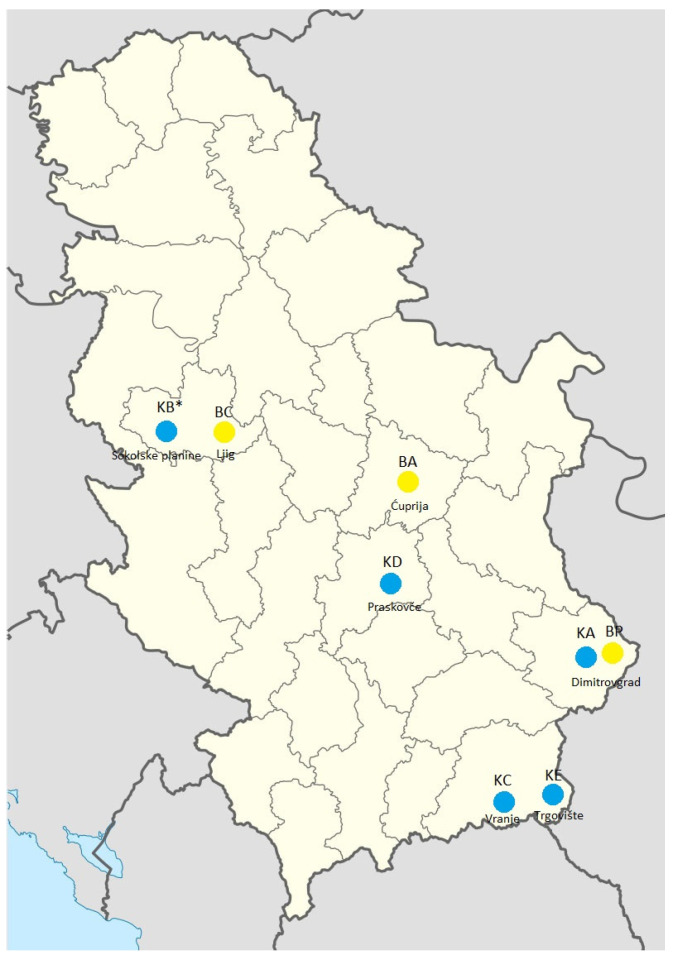
Sampling sites in Serbia. Bardoka sheep were sampled at sites BA-Ćuprija, BC-Ljig and BP-Dimitrovgrad, which are labeled with yellow circles. Karakachan sheep were sampled at sites KA-Dimitrovgrad, KB*-Sokolske planine (* considering the low number of animals at this site, they were analyzed together with animals from the flock at site KA), KC-Vranje, KD-Praskovče, and KE-Trgovište, which are labeled with blue circles.

In Serbia, Bardoka sheep are present in the municipality of Dimitrovgrad in the southern part of the country and at the sites Ćuprija and Ljig in central Serbia (Figure 2). According to the data from the herd book, the number of registered Bardoka sheep that were under production trait control was 107 in 2019, when the sampling for our study was initiated. This number increased to 289 in 2024.

#### 2.2.2. Karakachan Sheep

Karakachan sheep are present in Bulgaria, Greece, North Macedonia, and Serbia, where the breed is used for combined production traits (milk, meat, and wool). Karakachan sheep are considered one of the oldest European sheep breeds. The development of this breed is linked to the nomadic Karakachan people whose occurrence in the southeastern Balkan Peninsula dates back to the Middle Ages [44]. They practiced the most primitive type of livestock breeding, nomadic breeding, which is conservative and directed towards the selection of animals that can walk over long distances, graze on poorly accessible terrain, and survive in harsh and scarce environmental conditions [45]. Nomadic breeding has led to the development of a small hardy sheep whose extant rams have a height at withers of 50–55 cm and a live weight of 55–60 kg, while ewes have a height at withers of 45–50 cm and a live weight of 45–50 kg [11,12,45]. Karakachan sheep exhibit slow growth, high disease resistance, and a robust constitution with a well-developed body structure while maintaining low nutritional and housing requirements.

The coarse Karakachan sheep wool is up to 26 cm long and is typically gray-black and brown-black, rarely white [11,12,45]. The fleece is open and composed of spiky locks which do not curve. The wool covers all body parts and forms a tuft on the forehead, which is a typical feature of Karakachan sheep. The head is overgrown with wool up to above the eyes, and the face is covered with short, black hair that loses its shine and color as the sheep age. The belly is well developed and covered with wool so that the udder is often concealed in the wool. The head is relatively small and narrow, with a slightly wedge-shaped appearance from the front. In ewes, the head is almost always flat and the profile is rarely slightly convex. In rams, it is always distinctly convex. Rams are mainly horned, ewes rarely. The horns in rams are saber-shaped, very strong at the base, transversely furrowed, and curl into a spiral. The neck is of medium length and rather muscular, and the chest is of medium depth. The trunk is rather short with a compact body structure. The legs are of medium height, rather strong, and covered with hair, with hooves that are relatively small but very firm and always pigmented.

In Serbia, Karakachan sheep are present in the municipalities of Dimitrovgrad, Bosilegrad, Vranje, and Kruševac in the southern part of the country and at the Sokolske kolibe site in the central part of Serbia (Figure 2) where only animals with black wool are present (Figure 1D–F). According to the data from the herd book, the number of registered Karakachan sheep under production trait control was 176 in 2019, when the sampling for our study was initiated. This number increased to 345 in 2024.

### 2.3. Sampling and DNA Extraction

Sampling was carried out in the period from 2019 to 2021, and blood from the jugular vein of each animal was individually collected in tubes containing ethylenediamine tetra-acetic acid (EDTA) by a veterinarian. The tubes with blood were stored at −20 °C prior to the extraction of the total genomic DNA. In total, samples were collected from 230 animals, namely 105 Bardoka sheep (97 females, 8 males) from three flocks (localities BA, BC, and BP), and 97 Karakachan sheep (86 females, 11 males) from five flocks (localities KA, KB, KC, KD and KE) (Figure 2). At least one male per flock was sampled. Due to the low number of Karakachan sheep sampled from locality KB (six), these individuals were grouped with those from the flock at the locality KA for further analyses. This was justified because animals from the KB flock were purchased from the farmer maintaining the KA flock. Since no data on previous genetic exchange between KE and other Karakachan sheep flocks were available, this flock of small size was not merged with any other flock of this breed. We mainly sampled animals for which pedigree data were available for at least two generations, but also phenotypically adequate individuals for which pedigree data were incomplete or missing. Information on the underlying reasons for the presence of animals with known and unknown pedigree data is given in Supplementary Materials S1. For a comparison with autochthonous breeds, we used a commercial breed Ile-de-France (IDF) represented by 28 animals from a flock maintained at the commercial farm in locality Bogatić (25 females, 3 males).

The total genomic DNA was extracted from 100 μL of the blood of each individual, using the Zymo Research D3025 Quick-DNA miniprep kit (Zymo Research, Irvine, CA, USA), according to the manufacturer’s instructions. DNA yield and purity were determined spectrophotometrically, using the BioSpec-Nano (Shimadzu Europe, Duisburg, Germany).

### 2.4. Genotyping

Genotyping was performed with 14 nuclear microsatellites recommended by the International Society for Animal Genetics (ISAG) and the Food and Agriculture Organization of the United Nations (FAO UN) for sheep genotyping (Table 1), which were assembled into three panels for parallel PCR amplification. PCR amplification was conducted with a Type-it Microsatellite PCR kit (Qiagen, Hilden, Germany) following the manufacturer’s instructions. PCR reactions were performed with a thermocycler (Biometra, Analytic Jena, Germany) with the following cycling conditions: initial denaturation at 95 °C for 5 min, 28 cycles with denaturation at 95 °C for 30 s, annealing at 57 °C for 90 s, elongation at 72 °C for 30 s, and final extension at 60 °C for 30 min. Separation of PCR products was performed using capillary electrophoresis, using an 8-capillary ABI Prism 3100 Genetic Analyzer (Applied Biosystems, Inc., Foster City, CA, USA). GeneScan 500LIZ dye Size Standard (Thermo Fisher Scientific, Waltham, MA, USA) was used as a ladder. Fragment sizing was performed with GeneMapper ver. 4.0 (Applied Biosystems, Foster City, CA, USA).

### 2.5. Data Analyses

The obtained multilocus genotypes of all individuals were deposited in the public database Figshare [46]. The genotypes were first used to assess relatedness (r) among pairs of individuals with the triadic likelihood estimator (TrioML) implemented in the software package Coancestry v.1.0.1.9 [47]. TrioLM is expected to produce r values of zero for unrelated individuals, ~0.25 for second-order relatives (half-siblings or grandparent–grandchild), and ~0.5 for first-order relatives (full siblings or parent–offspring) [47]. For each studied breed, we assessed the pairwise relatedness of ewes and the pairwise relatedness of rams. Multilocus genotypes were also used to assess inbreeding values for each individual (I) estimated with the TrioML estimator implemented in the same software package.

Multilocus genotypes of unrelated females were used for assessing standard parameters of genetic diversity, namely the number of alleles per locus (Na), the effective number of alleles (Ae), private alleles (PAs), observed heterozygosity (H_O_), expected heterozygosity (H_E_), and fixation index (F_IS_), using the software package GenAlEx 6.5 [48]. Allelic richness (Ar) was computed based on rarefaction in HP-Rare [49], considering differences in sample size and a common number of 8 gene copies in the case of flocks (Ar8), and 48 gene copies (Ar48) in the case of breeds. The effective population size (Ne) was inferred based on the linkage disequilibrium method implemented in NeEstimator v2.1 [50], discarding alleles with frequencies < 0.02.

Genetic differentiation expressed via Wright’s F_ST_ index [51] was assessed between Bardoka, Karakachan, and IDF sheep and between flocks belonging to autochthonous populations using GenAlEx. The same software was used to estimate gene flow (Nm). Genetic structure was further studied by performing Principal Coordinate Analyses (PCoAs) based on the F_ST_ matrix carried out using GenAlEx. PCoAs were used to assess the genetic structure among three studied breeds, among flocks of both studied autochthonous breeds, as well as among all individuals, rams and ewes, from all three studied breeds. Genetic structure was also assessed using the Bayesian clustering method in STRUCTURE 2.3.4. [52] to determine the optimal number of independent genetic groups (K). In STRUCTURE simulation, a burn-in of 600,000 and run length of 600,000 iterations were specified, and ten independent runs for each of the assumed K = 1–10 were performed under the models of no admixture and independent allele frequencies. The ΔK method of Evanno et al. (2005) [53] implemented in StructureSelector [54] was used to determine the optimal number of genetic groups and to detect possible higher levels of hierarchical structure. The post-processing of results was carried out with CLUMPAK [55] implemented into the same software.

## 3. Results

### 3.1. Relatedness and Inbreeding Values of Individuals

**Figure 3:**
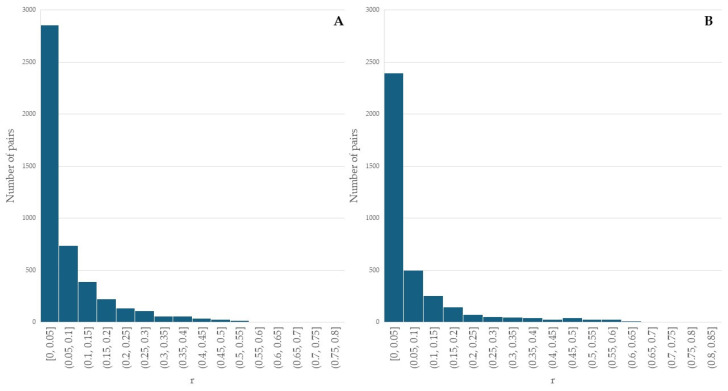
Pairwise relatedness of Bardoka and Karakachan ewes; (**A**) pairwise relatedness of Bardoka ewes; (**B**) pairwise relatedness of Karakachan ewes.

**Figure 4:**
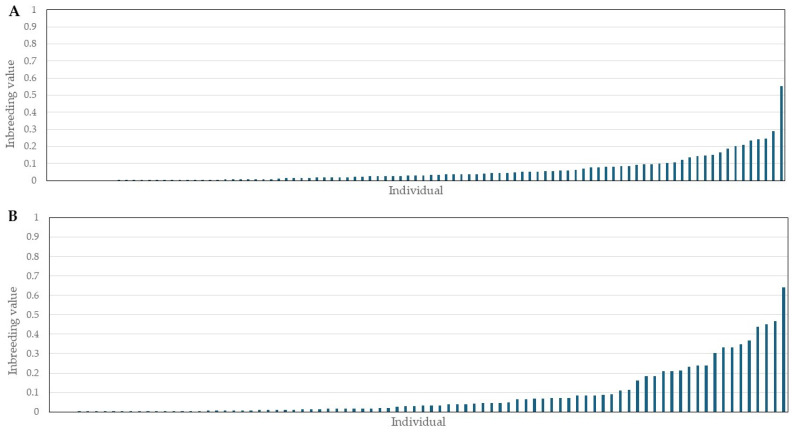
Inbreeding values of Bardoka and Karakachan ewes; (**A**) inbreeding values of Bardoka ewes; (**B**) inbreeding values of Karakachan ewes.

The pairwise relatedness (r) between 97 Bardoka ewes ranged from 0 to 0.759, with the majority of pairs (90.0%) having r ≤ 0.2, and 28 pairs (0.6%) having r ≥ 0.5, with an average of 0.06 (Figure 3A). The pairwise relatedness between 86 Karakachan sheep ewes ranged from 0 to 0.814, with the majority of pairs (90.0%) having r ≤ 0.2, and 79 pairs (2.1%) having r ≥ 0.5, with an average of 0.07 (Figure 3B). Although first-order relatives were not present in the same but in different flocks, it was necessary to exclude one individual from each of the Bardoka and Karakachan sheep pairs with r ≥ 0.5 to ensure the genetic analysis of unrelated individuals at the population level [56]. We used inbreeding values of individuals (I) as exclusion criteria, which in Bardoka ranged from 0 to 0.3 in all individuals with one exception (one individual had I = 0.554), and averaged 0.06 (Figure 4A). In the Karakachan sheep, eight individuals had 0.3 ≤ I ≤ 0.5 and one had I = 0.640, while the average I value was 0.09 (Figure 4B). An individual with the higher I value from a pair with r ≥ 0.5 was excluded and, in that way, we assembled a data set for all further analyses which included 77 Bardoka ewes and 61 Karakachan ewes (Table 2).

**Figure 5:**
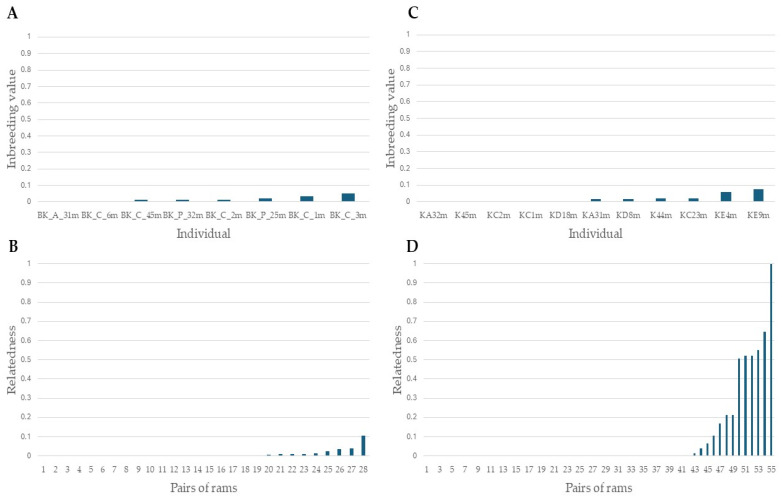
Inbreeding values and pairwise relatedness of Bardoka and Karakachan sheep rams; (**A**) inbreeding values of Bardoka rams; (**B**) pairwise relatedness of Bardoka rams; (**C**) inbreeding values of Karakachan sheep rams; and (**D**) pairwise relatedness of Karakachan sheep rams.

The inbreeding values in eight Bardoka rams were below 0.05 in all individuals, with an average I = 0.02 (Figure 5A). Their pairwise relatedness was up to 0.04 in 27 out of 28 pairs (96.4%), with only 1 pair with an r = 0.1 (3.6%). The average r of Bardoka rams was 0.01 (Figure 5B). The inbreeding in 11 Karakachan rams was <0.08 in all individuals, with an average I = 0.02 (Figure 5C). Their pairwise relatedness was <0.04 in 44 out of 55 pairs (80.0%), approximately 0.5 in 5 pairs (9.1%), and r = 1 in 1 pair (1.8%). The average relatedness of Karakachan sheep rams was 0.08 (Figure 5D).

### 3.2. Genetic Diversity

All parameters of genetic diversity in 77 Bardoka and 61 Karakachan sheep were relatively high and generally comparable between the breeds (Table 2). The number of alleles, effective number of alleles, number of private alleles, and estimates of observed heterozygosity were slightly higher in Bardoka sheep [152, 4.87 (±0.49), 31, 0.770 (±0.029) vs. 149, 4.61 (±0.40), 29, 0.767 (±0.022)], while allelic richness rarefacted to 48 gene copies and effective population size were slightly higher in Karakachan sheep [8.66, 56.0 (CI_95%_ 48.0, 66.2) vs. 8.39, 50.1 (CI_95%_ 44.3–57.0)]. The expected heterozygosity in Bardoka was 0.761 (±0.028) and 0.761 (±0.021) in Karakachan sheep. In the IDF flock of 25 females, all parameters of genetic diversity were slightly lower but comparable to values detected in Bardoka and Karakachan sheep. However, the effective population size was almost double in IDF [90.4 (CI_95%_ 50.5–311.7)], in comparison with the values obtained in Bardoka and Karakachan sheep. A statistically significant excess of heterozygotes was observed in Bardoka sheep (F_IS_ = −0.013 (±0.010)) and IDF (F_IS_ = −0.080 (±0.026)).

All parameters of genetic diversity in the BA, BC, and BP flocks of Bardoka were comparable, except for the effective population size, which was 53.4 (CI_95%_ 38.5–82.6) in BA, 38.6 (CI_95%_ 31.4–48.8) in BC, and 30.4 (CI_95%_ 23.7–41.0) in BP. A statistically significant excess of heterozygotes was observed in the BC [F_IS_ = −0.048 (±0.015)] and BP flocks [F_IS_ = −0.056 (±0.023)] (Table 2). Comparable values of parameters of genetic diversity were obtained for two Karakachan sheep flocks, namely KA and KC, which were also characterized by comparable sample sizes. However, lower values of all parameters of genetic diversity were obtained in the Karakachan sheep flocks KD and KE characterized by smaller sample sizes (nine and four individuals, respectively) (Table 2). A statistically significant excess of heterozygotes was observed in KC, KD, and KE [F_IS_ = −0.146 (±0.038), −0.071 (±0.035) and −0.180 (±0.093), respectively].

### 3.3. Genetic Structure

**Figure 6:**
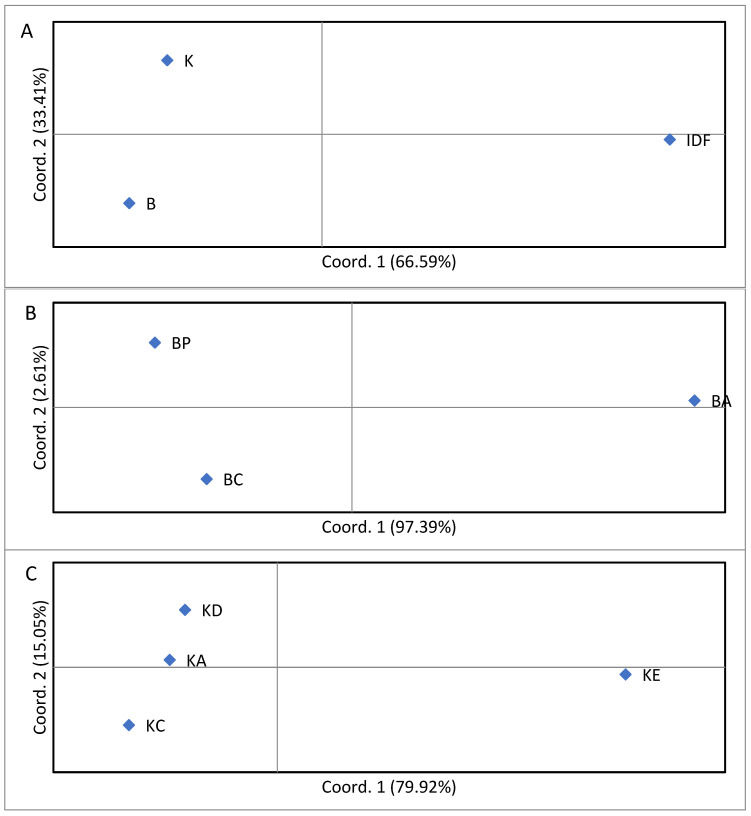
(**A**) PCoA with Bardoka, Karakachan, and Ile-de-France sheep used for a comparison; (**B**) PCoA with the BA, BC, and BP flocks of Bardoka; (**C**) PCoA with the KA, KC, KD, and KE flocks of Karakachan sheep.

The genetic differentiation between the Bardoka, Karakachan, and IDF sheep was low but statistically significant, as inferred from F_ST_ = 0.059 (*p* < 0.01). The genetic differentiation between IDF and each of the two autochthonous populations was low but statistically significant (ca. 0.05, *p* < 0.01), and lower but also statistically significant between Bardoka and Karakachan sheep (F_ST_ = 0.031, *p* < 0.01) (Table 3). The gene flow between the three populations was 4.32 ± 0.34 migrants per generation, and 13.90 ± 3.21 between Bardoka and Karakachan sheep. The pairwise population F_ST_ values provided in Table 3 were used for PCoAs, as presented in Figure 6A. The first two coordinates explained 66.59% and 33.41% of the molecular variation (in total 100%). IDF was separated from Bardoka and Karakachan sheep by the first coordinate, while the two autochthonous sheep populations were separated along the second coordinate.

**Figure 7:**
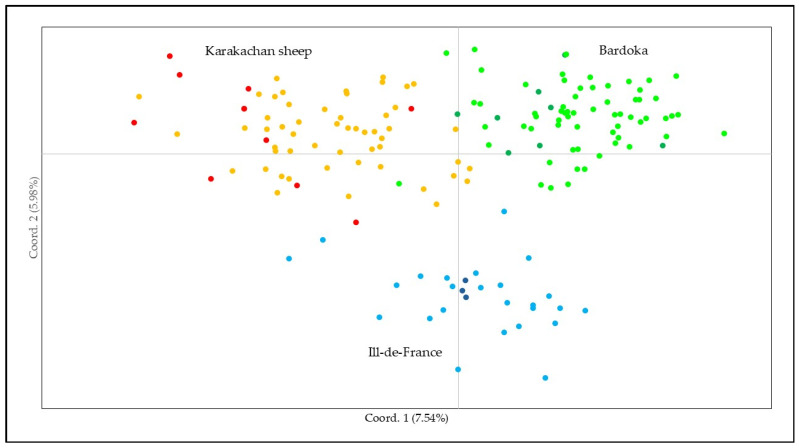
PCoA with Bardoka, Karakachan, and Ile-de-France ewes and rams; Bardoka rams are indicated with dark green circles, Bardoka ewes with light green circles, Karakachan rams with red circles, Karakachan ewes with yellow circles, Ile-de-France rams with dark blue circles, and Ile-de-France ewes with light blue circles.

Higher and statistically significant values of F_ST_ were detected between four Karakachan sheep flocks [0.118 (*p* < 0.01)] than between three Bardoka sheep flocks [0.019 (*p* = 0.002)]. Consequently, higher gene flow was observed between Bardoka flocks (Nm = 15.69 ± 2.57 migrants per generation) than between Karakachan flocks (Nm = 2.23 ± 0.26 migrants per generation). Pairwise flock F_ST_ values between Bardoka flocks and Karakachan flocks are presented in Table 4 and Table 5, respectively, and were used for PCoAs. In the PCoA plot with Bardoka flocks (Figure 6B), the first two coordinates explained 97.39% and 2.61% of the molecular variation, respectively (in total 100%). The BA flock was separated from the other two flocks by the first coordinate, while the BC and BP flocks were separated along the second coordinate. In the PCoA plot with Karakachan sheep flocks (Figure 6C), the first two coordinates explained 79.92% and 15.05% of the molecular variation, respectively (in total 94.97%). The KE flock was separated from the remaining flocks by the first coordinate, while the KA and KD flocks were separated from the KC flock along the second coordinate. The PCoA based on pairwise F_ST_ values between all studied animals, both ewes and rams, is provided in Figure 7. The first and second coordinates explained 7.54% and 5.98% of the molecular variation (in total 13.53%). Bardoka females and males were separated from Karakachan females and males by the first coordinate, and autochthonous breeds were separated from IDF along the second coordinate. In Bardoka and Karakachan sheep, males were scattered throughout the plot section occupied by the ewes from the corresponding breed, while IDF males occupied the central position in the plot section occupied by the IDF ewes.

**Figure 8:**
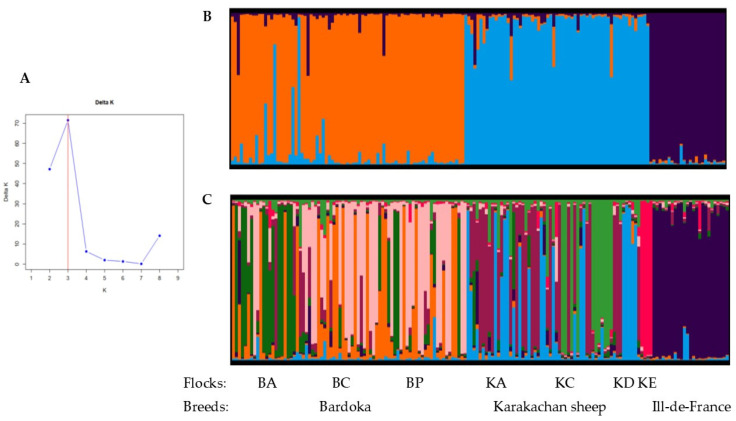
Outcomes of the Bayesian clustering with Bardoka, Karakachan, and Ile-de-France ewes; (**A**) the optimal number of gene pools according to the ΔK method of Evanno et al. (2005) [53]; (**B**) genetic structure presented with individual q values at K = 3; and (**C**) genetic structure presented with individual q values at K = 8.

The Bayesian clustering analysis was performed for K = 1–10. The strongest ΔK mode was observed at K = 3, indicating that the optimal number of genetic groups coincided with the number of studied breeds, but another ΔK mode of lower strength was observed at K = 8 (Figure 8A). According to Evanno et al. (2005) [53], the occurrence of several ΔK modes indicates substructuring within each of the detected groups, and the heights of each ΔK mode describe the strength of the genetic structure at each K. Clusters obtained in STRUCTURE analyses at K = 3 and K = 8, drawn using CLUMPAK, are presented in Figure 8B,C. At K = 3, animals belonging to Bardoka, Karakachan, and IDF were assigned to separate gene pools with a high average proportion of membership (qi ≥ 0.90). At K = 8, most of the animals belonging to Bardoka were assigned to three gene pools, and those belonging to Karakachan were assigned to four gene pools with a relatively high average proportion of membership (qi ≥ 0.80). However, the gene pools did not coincide with the studied flocks, although such a tendency has been observed; that is, individuals strongly assigned to one gene pool prevailed in the BA Bardoka flock, while in the BC and BP flocks, individuals strongly assigned to the second and third gene pools were present. Individuals strongly assigned to the fourth gene pool were observed in the KC flock of Karakachan sheep, and those assigned to the fifth gene pool prevailed in the KE flock. Individuals strongly assigned to the sixth and seventh gene pools were recorded in two flocks, KA and KD, but with differential abundances because the sixth gene pool prevailed in the KA flock and the seventh gene pool dominated in the KD flock. At K = 8, individuals with admixed genetic profiles were observed in both autochthonous breeds. All IDF animals remained strongly assigned to a single gene pool.

## 4. Discussion

Historically, the Balkan Peninsula and locally adapted sheep populations from this region played an important role in modeling the landscape of domestic sheep in Europe [1,2,3]. Extant autochthonous Balkan sheep breeds differ genetically from those in surrounding regions [3] and harbor rather high levels of genetic diversity [3,6,7] as well as genetic adaptive fitness traits [5,11,12], which may be highly valuable for future breeding efforts, especially in terms of global climate change [8]. Thus, the Balkan sheep breeds are regarded as a biodiversity reservoir whose conservation and sustainable use are imperative, especially in breeds with current critically low population sizes. In Serbia, this is the case with Bardoka and Karakachan sheep, which evolved from Pramenka (Zackel), the primary type of domestic sheep, under specific biogeographical and sociocultural conditions [5,9,10]. While these breeds have been studied at the molecular level in surrounding countries in which they are present, to the best of our knowledge, there is only one publication of this kind in which ca. 25 animals of each of these breeds originating from Serbia were studied [13]. In this study, we genetically analyzed a large proportion (>50%) of extant Bardoka and Karakachan sheep populations in Serbia and used genetic data to set the grounds for their genetically informed conservation and management, which are also applicable to other locally adapted breeds.

### 4.1. Relatedness and Inbreeding Values in Bardoka and Karakachan Ewes

Breeds characterized as critically endangered are at significant risk of inbreeding and genetic drift [20,21,22], which have negative effects on reproduction rate and productivity [23,24,25] and hamper the ability of the breed to cope with environmental changes [26,27]. Therefore, the estimation and management of the inbreeding values of individuals, traditionally inferred from the pedigree data [23,57,58], are important for the successful conservation and long-term survival of such (and other) breeds. However, in sheep and goats, pedigree-based inbreeding values can be severely underestimated [57,59] for several reasons, such as incomplete and/or missing data [38], extensive farming [39], and creation of mating groups with the simultaneous presence of more males [37]. A suitable alternative is the usage of genetic/genomic-based estimates of inbreeding values of individuals [37,60,61,62], which can also be integrated with the traditional approach to provide more accurate estimates of the relationship between individuals [60]. Considering the incomplete pedigree data for endangered Bardoka and Karakachan sheep in Serbia (Supplementary Material S1), we relied on genetic data to assess the relatedness at the population level and inbreeding values of the studied individuals.

Although a similar proportion of unrelated ewe pairs (90.0%, r ≤ 0.2) was observed in both breeds, the average pairwise relatedness among 86 Karakachan ewes was higher than that among 97 Bardoka ewes (0.07 vs. 0.06, respectively). Expectedly, higher average inbreeding was observed in Karakachan sheep ewes than in Bardoka ewes (0.09 vs. 0.06, respectively). It is worth mentioning that I > 0.3 was present in 10.5% of Karakachan sheep ewes and only in 1.0% of Bardoka ewes. Our findings demonstrate that according to expectations, increased relatedness and inbreeding values of individuals were observed in the breed that experienced a more severe population decline, the Karakachan sheep. Nonetheless, the average inbreeding in this breed was lower than that reported for some purebred sheep populations which ranged from 2% to 5% [23,63,64].

It is important to mention that the first-order relatives were not present in the same flock but were observed at the population level in both breeds. Although they were excluded appropriately for the subsequent genetic analyses [56], their presence, which was four times higher in Karakachan sheep than in Bardoka (2.1% vs. 0.06%, respectively), may indicate somewhat different flock-rebuilding strategies in the studied breeds; that is, it is possible that flock rebuilding in Karakachan sheep is accomplished not only by self-replacement but also by purchasing ewes either as ewe lambs, yearlings, or adult ewes. In that way, a larger proportion of full siblings and/or mother–offspring pairs distributed across different flocks would be present in a population instead of being used for meat production (i.e., being sold to slaughterhouses). Although such management is justified to maintain/increase the number of animals in terms of low population size, it should be performed carefully to ensure the maintenance of low-inbred and unrelated animals in flocks. Flock-rebuilding strategies in autochthonous breeds in developing countries are occasionally driven by goals that are not as sophisticated as in the case of high-productive commercial breeds [65]. Furthermore, in extensively farmed breeds, some flock-rebuilding strategies such as minimizing losses by improving fertility and lamb survival, increasing reproductive potential through higher ovulation rates, early-age mating, and multiple matings per year [66,67] are simply logistically challenging and/or economically unsustainable when considering the cost–benefit ratio.

### 4.2. Genetic Diversity in Bardoka and Karakachan Sheep

Despite the extant critically low population size of both Bardoka and Karakachan sheep in Serbia, all parameters of genetic diversity in both breeds were relatively high (Table 3). The number of alleles at 14 microsatellite loci was comparable in Bardoka and Karakachan sheep (152 and 149, respectively), and the same holds for the effective number of alleles (4.87 ±0.49 in Bardoka and 4.61 ±0.40 in Karakachan sheep) and allelic richness rarefacted to 48 gene copies (8.33 in Bardoka and 8.39 in Karakachan sheep). Both breeds comprised ca. 30 private alleles each, while a low but statistically significant excess of heterozygotes was observed in Bardoka [F_IS_ = −0.013 (±0.010)] but not in Karakachan sheep [F_IS_ = −0.010 (±0.014)]. The values of expected heterozygosity were similar in both breeds, namely 0.761 (±0.028) in Bardoka and 0.761 (±0.021) in Karakachan sheep. The values were comparable to those reported by Ćinkulov et al. (2008) [13] for the same breeds from Serbia (H_E_ = 0.756 in Bardoka and H_E_ = 0.739 in Karakachan sheep), obtained using the same type of molecular markers for the analysis of ca. 25 individuals per breed. They were also comparable to those based on microsatellite variability studied in the breeds in question from neighboring countries, namely Bardoka from Albania (H_E_ = 0.76) [68] and Montenegro (H_E_ = 0.743) [17], and Karakachan sheep from Bulgaria (H_E_ = 0.78 [18,19] and H_E_ = 0.77) [69,70]. Generally comparable H_E_ values have been reported in other locally adapted breeds from the Balkans, for example, seven Western Balkans Pramenka types [13], twelve eastern Adriatic and western Dinaric native breeds [71], seven local breeds from Bosnia and Herzegovina and Croatia [72], four Slovenian and Croatian native breeds [73], Bulgarian autochthonous breeds [18,19,69,70], and local Greek breeds [74,75]. Regarding the commercial sheep breeds, it is worth mentioning IDF for which our estimates of expected heterozygosity were somewhat higher than those reported by Leroy et al. (2015) [76] (0.763 ± 0.031 vs. 0.66, respectively).

The patterns of change in effective population size in breeds, traditionally inferred from pedigree data [77], are important indicators of the success of conservation [78]. Over the past five years, the Ne in studied autochthonous breeds varied from 19.07 in 2019 to 42.33 in 2024 in Bardoka and from 41.25 in 2019 to 35.06 in 2024 in Karakachan sheep as inferred from the available pedigree data (data obtained from the Institute of Animal Husbandry, unpublished). Our estimates of Ne values were somewhat higher, namely 50.1 (CI_95%_ 44.3, 57.0) in Bardoka and 56.0 (CI_95%_ 48.0, 66.2) in Karakachan sheep, and concordant with expectations for breeds characterized as critically endangered based on the FAO risk status criteria [28]. In the case of flocks, the Ne value in the Karakachan sheep flock KA, represented by 31 individuals, was almost double in comparison with that recorded in the KC flock, which comprised almost half as many individuals. A similar trend, with larger Ne values in larger flocks, was not observed in Bardoka, in which the Ne was halved in one of the two flocks of similar size [BA: Ne = 53.4 (CI_95%_ 38.5, 82.6); BC: Ne = 38.6 (CI_95%_ 31.4, 48.8)]. Considering the expected positive correlation between the census size (Nc) and Ne values estimated using the linkage disequilibrium method [78], a higher Ne would be expected in larger flocks. As that was not the case for the BA and BC Bardoka flocks, it is possible that a larger proportion of older ewes that do not contribute to the formation of the next generation was present in the BC flock. This would imply that improvement of the flock-rebuilding strategy is required in this Bardoka flock. Regarding the levels of genetic diversity in Bardoka and Karakachan sheep flocks, all parameters of genetic diversity were more or less comparable to those observed at the population level in both breeds, with the exception of two Karakachan sheep flocks, KD and KE, represented by a lower number of individuals in which, consequently, lower levels of genetic diversity were indicated by all parameters (given the low flock size, parameters of genetic diversity in these flocks should be taken with caution).

Altogether, based on the observed values of genetic diversity parameters, it appears that the studied Bardoka and Karakachan sheep from Serbia have good prospects for long-term survival if flock-rebuilding strategies can be improved and further upgraded through the usage of genetic/genomic data that can provide guidance for management practices. However, it is worth mentioning that past admixture within local populations and introgression of non-native gene pools into the local ones, whose extent is unknown [5,19], must have affected our estimates of genetic diversity in Bardoka and Karakachan sheep to a certain extent. This is because such processes are known to contribute towards the introduction of new alleles into populations and to increased levels of genetic diversity [26,56]. Further studies that analyze Bardoka and Karakachan sheep in a broader context and with additional genetic or genomic markers [79,80,81] are required to assess breed-specific portions of the genome, distinguish purebred from admixed/introgressed individuals, and refine levels of genetic diversity in these breeds.

### 4.3. Genetic Differentiation Between and Within Bardoka and Karakachan Sheep

Recent genomic studies, which investigated some of the Pramenka-type breeds in a broader context, revealed a considerable genetic overlap of local Balkan sheep populations [3], which is indicative of their incomplete genetic differentiation. Alternatively, the observed pattern may be at least partly a result of the indiscriminate past management practices characterized by intentional or unintentional uncontrolled crossbreeding [17,18,19]. It is well known that geographic isolation contributes to genetic isolation over time [82,83], while gene flow counteracts differentiation from selection and drift when it occurs during divergence or on secondary contact [84,85,86]. Our data further support the observed pattern of low genetic differentiation among local Balkan sheep breeds regardless of the underlying reasons for such a pattern. This is because low genetic differentiation of ca. 5% (*p* < 0.01) was observed between a commercial breed used for comparisons, IDF, and each of the two autochthonous breeds studied, while lower genetic differentiation of 3.1% (*p* < 0.01) was found between Bardoka and Karakachan sheep. The latter value is comparable to that reported previously for different Pramenka-type breeds, which ranged from ca. 2% to ca. 5% [13,18], as well as to those observed between native Balkan sheep breeds [21,69,70] and other local breeds found elsewhere, for instance, 3.6% between five Moroccan sheep breeds [87], 3.8% to 4.4% between Algerian breeds [88,89], and 3.0% between six Tunisian sheep breeds [90]. However, the genetic differentiation was lower in comparison to that observed among some other breeds, such as some Austrian breeds [91], Romanian breeds [92], Spanish breeds [93,94,95,96], Portuguese breeds [97], Nigerian breeds [98], Turkish breeds [99], 57 European and Middle Eastern native and commercial breeds [6], and Baltic breeds [100], all of which were estimated based on microsatellite variability. It is worth mentioning that lower F_ST_ estimates were reported as well, for instance, 1.1% between some Albanian native breeds [68] and 1.7% between some Tunisian breeds [101]. In our study, genetic differentiation of phenotypically different Bardoka and Karakachan sheep was further supported by their clear separation from each other in the PCoA along the second coordinate, which explained as much as 33.41% of molecular variation (Figure 6A).

Insights into the within-breed genetic structure of Bardoka and Karakachan sheep were obtained as well by assessing the levels of genetic differentiation of their flocks. We found higher and statistically significant genetic differentiation between four Karakachan sheep flocks (11.8%, *p* < 0.01) than between three Bardoka flocks, which was lower but also statistically significant (1.9%, *p* < 0.01). Interestingly, Odjakova et al. (2023) [18] also reported a higher level of genetic differentiation between two Karakachan sheep flocks than between the flocks of the Rhodopean tsigai and the Middle Rhodopean sheep. Furthermore, Mihailova et al. (2023) [19] also observed genetic heterogeneity of five Karakachan sheep flocks. This may be explained by the more prominent genetic drift effects in Karakachan sheep flocks than in the flocks of other breeds, possibly due to the more severe reduction in the population size of this breed in comparison with other breeds as well as low gene flow among flocks [29,102]. Alternatively, this may also be explained by differential genetic input from admixture and/or introgression into different flocks [95.96]. We found relatively high gene flow between Bardoka flocks (15.69 ± 2.57 migrants per generation) and seven times lower gene flow between Karakachan sheep flocks (2.23 ± 0.26% migrants per generation). Further investigations revealed that two Bardoka flocks, BC and BP, displayed genetic similarity as they were separated from the third flock, BA, along the first axis in the PCoA, which explained 97.39% of the molecular variation (Figure 6B). In the case of the Karakachan sheep, one flock, KE, was genetically distinct from other flocks, among which the KA and KD flocks were genetically more similar compared to the KC flock (Figure 6C). The observed pattern reflects recent activities related to the exchange of animals among studied flocks exceptionally well, which was present between the BC and BP Bardoka flocks, and the KA and KD Karakachan sheep flocks. Thus, it appears that the current within-breed genetic structure of the Karakachan sheep as well as Bardoka was modeled by rather complex past and present genetic and demographic processes and management practices.

The between-breed and within-breed genetic structure inferred from the values of Wright’s F_ST_ parameter and PCoA was fully concordant with that observed using the Bayesian clustering method of Pritchard et al. (2000) [52]. The optimal number of three gene pools coincided with the number of studied breeds, with a rather high percentage of animals strongly assigned to the appropriate breed (Figure 8B). However, we observed a higher level of hierarchical structure as well, with eight gene pools. Although the number of eight gene pools coincided with the number of studied flocks (three Bardoka flocks, four Karakachan flocks, and the IDF flock), a straightforward pattern of one flock/one gene pool was not observed. Instead, we found that the distribution of animals strongly assigned (qi ≥ 0.80) to different gene pools reflects the pattern of recent gene flow described above. That is, at K = 8 in Bardoka, one gene pool was predominant in the BA flock, and two gene pools were found in two flocks, BC and BP, among which recent gene flow was present. In Karakachan sheep, different gene pools dominate in each of the two flocks, KC and KE, while the other two gene pools observed in this breed were distributed in the two remaining flocks, KA and KD. Expectedly, all IDF animals were strongly assigned to a single gene pool, which indicates that this breed is genetically coherent.

The applied Bayesian method for genetic structure assessment was used in several recent studies that analyzed microsatellite variability in locally adapted sheep populations from the Balkans. However, the outcomes were heterogeneous even for the same breed. For instance, seven local Montenegrin breeds were generally well separated from each other at K = 7, but with a prominent substructure in Bardoka [17], as observed in our study as well. However, this breed was not clearly distinguished from the other two studied native breeds from Albania based on the Bayesian clustering method [103]. Karakachan sheep from Bulgaria studied with two other native breeds were genetically distinct but substructured, while the other two studied native breeds were highly admixed [18]. In addition, each of the seven Bulgarian native breeds including Karakachan sheep was well distinguished into separate gene pools in the study of Hristova et al. (2014) [69]. Mihailova et al. (2023) [19] observed a rather complex and heterogeneous genetic structure in 12 native Bulgarian breeds as they found that some breeds were genetically coherent as all of their flocks belonged to a single distinct gene pool, other breeds were substructured as some of their flocks belonged to one gene pool while other flocks belonged to another gene pool (e.g., Karakachan sheep), while some breeds were highly admixed. This highlights the need for further studies, preferably at the regional level, which will employ additional genetic and genomic markers and analyze these breeds together with other local breeds. That, and their comparisons with commercial breeds known for their introgression into local breeds, such as Merino [5], will help us gain insight into the apparently rather complex genetic structure of Bardoka and Karakachan sheep, including the levels of admixture and introgression needed to assess the authentic portions of the genomes of local breeds.

### 4.4. Genetic Layout of Bardoka and Karakachan Ram Populations

The genetic contribution of an individual ram to the next generation is greater than that of an individual ewe, as fewer rams are retained each year in flocks. Therefore, the inaccurate selection of rams is magnified in the next generation to a greater extent than in the case of inaccurately selected ewes [104]. When the population size of a breed becomes critically low, the limited number of suitable rams for mating poses a challenge to maintaining genetic diversity and integrity and preventing inbreeding. In such situations, the use of optimized selection methods, such as linear programming (LP) and optimum contribution selection (OCS), can be considered as a strategy to increase genetic gain while controlling the level of inbreeding. Studies have shown that the LP method can significantly improve genetic gain, especially for traits with high heritability, although it can lead to higher levels of inbreeding. In contrast, the OCS method effectively controls inbreeding while achieving sustainable genetic gain during long-term selection [105,106]. Considering the decline in the population size of both Bardoka and Karakachan sheep in Serbia over the past few decades, which was more pronounced for the latter breed, we directed our attention to the ram population of both breeds as well. A low average inbreeding value of 0.02 was observed in rams of both breeds and the maximal inbreeding recorded in a single ram was 0.08 in Karakachan sheep and 0.05 in Bardoka. Our findings are rather important because they indicate that the breeding and selection of low-inbred rams were generally successful in both breeds. Such low-inbred rams are thus suitable for producing the next generation in both breeds.

Considering the pairwise relatedness of the studied Bardoka and Karakachan sheep rams, however, similar conclusions cannot be drawn in the case of the Karakachan sheep. We found that the average relatedness of Karakachan rams was eight times higher than that in Bardoka (0.08 vs. 0.01, respectively), as well as that the Karakachan rams were first-order relatives in 9.1% of pairwise comparisons. In this breed, we even recorded one pair of monozygotic twins (r = 1). That was not evident from the pedigree data, which erroneously described monozygotic twins as full siblings whose relatedness is ca. 0.5. The natural occurrence of monozygotic twins in sheep is less than 1% and the same holds for triplets [107,108]. However, the available data on the frequency of like-sex twins are unambiguous [108]. On the other hand, our findings highlight the utility of genetic data in providing information relevant to herd management and conservation regarding the usage of adequate rams for mating in different flocks to manage genetic diversity and inbreeding. This is important because their genetic layout permanently affects the genetic structure of the offspring, flocks, and ultimately the breed itself [104]. On the other hand, they indicate that the severe decline in the population size affected the ram population of Karakachan sheep to a larger extent than that of Bardoka.

We also found that contrary to the studied IDF rams, Bardoka and Karakachan ram populations were genetically rather heterogeneous. IDF rams, which were unrelated (r = 0) and characterized by no inbreeding (I = 0), were grouped together in the center of the PCoA plot section occupied by the ewes of this breed while rams of both Bardoka and Karakachan sheep were dispersed within the space occupied by the ewes of the corresponding breed (Figure 7). The observed genetic heterogeneity of rams of the studied autochthonous breeds, which was more pronounced for the Karakachan sheep than for Bardoka, may reflect the differential genetic input of other local and/or non-native breeds that were not used in our study. As previously mentioned, admixture and introgression of exotic gene pools into the local breeds in the Balkans were common in the past [5,18,19]. Further studies comparing a larger number of local and non-native breeds at the regional level using high-resolution molecular markers are required to provide better insights into the genetic profile and breeding value of rams in both Bardoka and Karakachan sheep. Such studies, which will investigate ram populations as well, will provide insights into whether Bardoka and Karakachan rams from neighboring countries should be considered for introduction into flocks of these breeds from Serbia to maintain the genetic integrity of breeds and avoid inbreeding. This is particularly important in the case of Karakachan sheep, for which our data imply that the methods of optimized selection may be needed in the near future. However, this may be a challenging task because of the low number of living animals of Karakachan sheep present at the regional level [45]; see also [79,109].

## 5. Conclusions

Our data demonstrate that population decline has had more profound negative effects on Karakachan sheep than on Bardoka. Nonetheless, both breeds have rather high levels of genetic diversity in their populations despite their current low population size. The observed within-breed genetic structure should be taken into consideration for management practices and conservation, as well as the genetic layout of rams, which is important for maintaining high levels of genetic diversity, genetically pure and noninbred individuals. Therefore, our data can serve as a guideline for setting rules for management practices which, when implemented in the scope of conservation programs, would lead to genetically informed conservation of Bardoka and Karakachan sheep in Serbia. Although this would increase their prospects for long-term survival, further studies, preferably at the regional level, providing additional genetic markers at the genomic level, are required to better understand the overall genetic status of Bardoka and Karakachan sheep breeds. Their comparison with other local and commercial breeds will help us gain insight into the levels of admixture and introgression in each animal. This is important for maintaining the genetic integrity of breeds by delineating animals that should remain in conservation programs from those that should be removed. Studies on the genetic basis of adaptive fitness traits will shed more light on functional genetic diversity in these breeds, which may enable genomic selection and improvement of the production and productivity of these sheep. The establishment and/or improvement of available gene banks, in which genetic and reproductive material is stored in adequate conditions and is used for research, breeding, and ultimately for breed restoration, are other aspects that can be expected to further facilitate the long-term survival of the breeds in question.

## Figures and Tables

**Table 1:** Characteristics of 14 nuclear microsatellites used for genotyping in Bardoka, Karakachan, and Ile-de-France sheep.

	Locus	Primer Sequence 5′-3′	Product Size (bp)	Panel No.
1	CSRD247	F: GGACTTGCCAGAACTCTGCAAT R: CACTGTGGTTTGTATTAGTCAGG	209–255	P1
2	ETH225	F: GATCACCTTGCCACTATTTCCT R: ACATGACAGCCAGCTGCTACT	131–157	P3
3	ILST87	F: AGCAGACATGATGACTCAGC R: CTGCCTCTTTTCTTGAGAGC	137–173	P2
4	INRA006	F: AGGAATATCTGTATCAACCGCAGTC R: CTGAGCTGGGGTGGGAGCTATAAATA	110–132	P3
5	INRA063	F: ATTTGCACAAGCTAAATCTAACC R: AAACCACAGAAATGCTTGGAAG	169–201	P3
6	MAF65	F: AAAGGCCAGAGTATGCAATTAGGAG R: CCACTCCTCCTGAGAATATAACATG	123–137	P2
7	MAF70	F: CACGGAGTCACAAAGAGTCAGACC R: GCAGGACTCTACGGGGCCTTTGC	124–166	P2
8	McM042	F: CATCTTTCAAAAGAACTCCGAAAGTG R: CTTGGAATCCTTCCTAACTTTCGG	87–107	P2
9	OarFCB20	F: AAATGTGTTTAAGATTCCATACAGTG R: GGAAAACCCCCATATATACCTATAC	95–120	P1
10	OarFCB48	F: GAGTTAGTACAAGGATGACAAGAGGCAC R: GACTCTAGAGGATCGCAAAGAACCAG	125–177	P1
11	SPS113	F: AAAGTGACACAACAGCTTCTCCAG R: AACGAGTGTCCTAGTTTGGCTGTG	126–154	P1
12	SRCRSP8	F: TGCGGTCTGGTTCTGATTTCAC R: CCTGCATGAGAAAGTCGATGCTTAG	183–249	P3
13	TCRVB6	F: GAGTCCTCAGCAAGCAGGTC R: CCAGGAATTGGATCACACCT	217–255	P3
14	TGLA53	F: GCTTTCAGAAATAGTTTGCATTCA R: ATCTTCACATGATATTACAGCAGA	140–163	P3

**Table 2:** Parameters of genetic diversity in Bardoka, Karakachan, and Ile-de-France sheep.

Breed * Flock	N	Na	Ae	Ar_48_ Ar_8_	Ne (CI_95%_)	PA	H_O_	H_E_	F_IS_
**Bardoka**	**84**	**152**	**4.87 (±0.49)**	**8.39**	**50.1 (44.3, 57.0)**	**31**	**0.770 (±0.029)**	**0.761 (±0.028)**	**−0.013 (±0.010)**
BA	25	118	4.29 (±0.36)	4.22	53.4 (38.5, 82.6)	7	0.731 (±0.030)	0.742 (±0.024)	0.016 (±0.017)
BC	35	123	4.76 (±0.47)	4.33	38.6 (31.4, 48.8)	7	0.794 (±0.026)	0.759 (±0.026)	−0.048 (±0.015)
BP	24	110	4.51 (±0.46)	4.22	30.4 (23.7, 41.0)	2	0.777 (±0.044)	0.736 (±0.038)	−0.056 (±0.023)
**Karakachan sheep**	**62**	**149**	**4.61 (±0.40)**	**8.66**	**56.0 (48.0, 66.2)**	**29**	**0.767 (±0.022)**	**0.761 (±0.021)**	**−0.010 (±0.014)**
KA	31	128	4.39 (±0.33)	4.27	66.8 (47.4, 106.7)	11	0.772 (±0.024)	0.754 (±0.019)	−0.027 (±0.030)
KC	18	95	3.84 (±0.27)	3.97	31.7 (21.6, 53.7)	3	0.825 (±0.035)	0.720 (±0.022)	−0.146 (±0.038)
KD	9	81	3.74 (±0.45)	3.99	14.8 (18.2, 35.9)	1	0.714 (±0.049)	0.672 (±0.046)	−0.071 (±0.035)
KE	4	39	2.28 (±0.21)	2.79	- **	3	0.589 (±0.089)	0.498 (±0.057)	−0.180 (±0.093)
**Ile-de-France**	**25**	**95**	**3.90 (±0.44)**	**6.79**	**90.4 (50.5, 311.7)**	**10**	**0.763 (±0.031)**	**0.707 (±0.026)**	**−0.080 (±0.026)**

* Parameters of genetic diversity in breeds were calculated at the population level, i.e., by using multilocus genotypes of individuals from each of the three studied breeds, and not as a total/average of estimates given for flocks of a breed in question. Data provided in bold refer to breeds; data not given in bold refer to flocks. ** It was not possible to assess accurate Ne in the KE Karakachan sheep flock due to the small sample size. N—sample size; Na—number of alleles; Ae—effective number of alleles; Ar_48_—allelic richness in breeds rarefacted to 48 gene copies; Ar_8_—allelic richness in flocks rarefacted to 8 gene copies; Ne—effective population size with 95% Confidence Interval (CI_95%_); PAs—number of private alleles; H_O_—observed heterozygosity; H_E_—expected heterozygosity; and F_IS_—inbreeding coefficient.

**Table 3:** Pairwise breed F_ST_ values between Bardoka, Karakachan, and Ile-de-France sheep.

Bardoka	Karakachan Sheep	Ile-de-France	
0.000			Bardoka
0.031	0.000		Karakachan
0.052	0.051	0.000	Ile-de-France

**Table 4:** Pairwise flock F_ST_ values among the Bardoka flocks BA, BC, and BP.

BA	BC	BP	
0.000			BA
0.016	0.000		BC
0.019	0.009	0.000	BP

**Table 5:** Pairwise flock F_ST_ values among the Karakachan flocks KA, KC, KD, and KE.

KA	KC	KD	KE	
0.000				KA
0.024	0.000			KC
0.032	0.048	0.000		KD
0.122	0.146	0.139	0.000	KE

## Data Availability

The original data presented in the study are openly available in Figshare at https://doi.org/10.6084/m9.figshare.28330118.

## Supplementary Materials

The following supporting information can be downloaded at https://www.mdpi.com/article/10.3390/ani15091193/s1, Text S1: Serbian program for conservation of autochthonous animal genetic resources and its implementation.
