# Supplementary material for: Towards Genetically Informed Conservation of the Bardoka and Karakachan Sheep Breeds Autochthonous to Serbia
Source: Animals (Basel). 2025 Apr 23;15(9):1193. doi: 10.3390/ani15091193 (PMC12071161; doi:10.3390/ani15091193)
Supplement: Supplementary file 1 [file animals-15-01193-s001.zip › animals-3501833-supplementary.pdf]

Supplementary material S1. Serbian program for conservation of autochthonous animal genetic resources and its implementation

Since the breeding programs and strategies for conservation of autochthonous animal genetic resources are country-specific, we provide a short description of those applied in Serbia which are defined in several legal acts, as well as the means of their implementation. There are two Main Breeding Organization that maintain the livestock herd books with pedigree data. The Institute of Animal Husbandry (IAH) is responsible for the registration of animals based on the data obtained from Regional and Local Breeding Organizations that cover the central and southern part of the country while the Faculty of Agriculture, University of Novi Sad, covers the northern part of the country, Vojvodina. Only the registered animals, which are under the jurisdiction of these two Main Breeding Organizations, are included into the conservation programs based on de-fined breeding programs, and are entitled to subsidies which, however, differ for commercial and autochthonous breeds.

In the case of autochthonous breeds, animals are classified into two categories depending on the availability of pedigree data. Animals registered in the so-called main part of the herd book have documented pedigrees for at least two generations and are assigned a so-called HB registration number. They are under production traits control and are eligible for the maximum subsidies, which include support based on their HB status and support based on their classification as autochthonous genetic resources. Animals registered in the additional part, the so-called reserve book, are given an RB registration number because they don't have documented pedigree data but have been evaluated as typical of the breed by the Main Breeding Organizations. These animals are only eligible for partial subsidies, namely those granted for autochthonous genetic resources. If farmers subject RB-registered animals to two controlled mating cycles with HB-registered animals, their second-generation offspring receives HB registration and is thus eligible for the maximum subsidies. Since subsidies are important for farmers who maintain autochthonous breeds typically characterized by low productivity, it is in their own interest not only to provide RB registration numbers to unregistered animals, which are still pre-sent in rural parts of the country, but also to perform their mating with those that have HB registration numbers because their offspring will be entitled to maximal subsides.

The approach described was established 15 years ago to systematically introduce into the registration system all the animals belonging to autochthonous breeds. Its beneficial effects are already evident, because since established, it enabled an increase in number of registered animals of autochthonous breeds, in particular those with HB registration numbers. Another important aspect of the system described is the fact that it tends to avoid

uncontrolled crossing, which was rather common in the past, and that enables maintaining the genetic integrity of autochthonous breeds.
